# Clinical trials for cerebellar ataxia

**DOI:** 10.1007/s00415-022-11088-w

**Published:** 2022-04-15

**Authors:** Benjamin E. Schroeder, Neil P. Robertson

**Affiliations:** Department of Neurology, Division of Psychological Medicine and Clinical Neuroscience, Cardiff University, University Hospital of Wales, Heath Park, Cardiff, CF14 4XN UK

## Introduction

Cerebellar ataxias comprise a heterogeneous group of sporadic and inherited neurodegenerative diseases with both cerebellar and non-cerebellar features including extrapyramidal weakness and reduced cognition. A number of pathogenic models have been proposed to explain these deficits, but no pharmacological Disease Modifying Therapies (DMTs) are currently available. In this month’s journal club, we explore three recent clinical trials which have focused on these disorders.

The first trial investigates riluzole in spinocerebellar ataxia type 2, the second and third explore acetyl-dl-leucine and cerebello-spinal stimulation in a wider group of participants with varying causes of cerebellar ataxia.

## Safety and efficacy of riluzole in spinocerebellar ataxia type 2 in France (ATRIL): a multicentre, randomised, double-blind, placebo-controlled trial

Spinocerebellar ataxia type 2 (SCA-2) is a rare, genetic, cerebellar ataxia which can present in an amyotrophic lateral sclerosis-like phenotype. ATRIL investigated whether a 50 mg twice daily riluzole regime (a medication licensed for ALS) against placebo for one year, would improve the proportion of patients with a one-point reduction in SARA (Scale for Assessment and Rating of Ataxia). 45 patients were recruited between January 2018 and June 2019 from across France into a randomized, double-blind, placebo-controlled trial. Physiotherapy was standard care. Baseline characteristics were similar: median age 42 years (IQR: 36, 57); 51% female; with moderate disease (median SARA score—13.5 (IQR: 9.5, 16.5)), and disease duration [median 11 years (IQR: 6, 16)].

ATRIL failed to demonstrate superiority to placebo in its primary outcome (riluzole: *n* = 7/22, 32%; placebo: *n* = 9/23, 39%) with broad confidence intervals [mean difference of − 10.3% (95% CI − 37.4% to 19.2%, *p* = 0.75)]. With riluzole there was a median 0.5 SARA point worsening (IQR: − 1.5, 1.5) and placebo 0.3 points (IQR: − 1.0, 2.5) which was not significant (*p* = 0.70). Composite Cerebellar Functional Severity score (CCFS) was also included as SARA can be vulnerable to rater- and patient-factors. The CCFS was worse in the riluzole group (0.055, IQR: 0.014, 0.086) than in placebo (0.004, IQR: − 0.040, 0.020) *p* = 0.0050. Compliance was reasonable [riluzole: 94% (SD 9.75); placebo: 94% (SD 5.77)]. Incidence of adverse events (AEs) were similar (riluzole: 73%, placebo 83%, *p* = 0.49) with four significant adverse events in the placebo group for which one patient withdrew (depression), and none in the riluzole group.

### Comment

ATRIL is the largest RCT of SCA-2 patients to date, and although it failed to show efficacy for riluzole, it presents some interesting insights for planning future trials. These include inclusion of an objective, validated measure for physical therapy to aid baseline characteristics, the value of addressing placebo effects and clarity on patient populations when identifying potential DMTs.

Lancet Neurol. 2022 Mar;21(3):225–233.

## Safety and efficacy of acetyl-dl-leucine in certain types of cerebellar ataxia

Acetyl-dl-leucine has been used in acute vertigo and suggested to be of benefit in several small case-series in ataxia. This trial used a randomized, double-blind, placebo-controlled, two-treatment two-period cross-over design, with a 4-week washout period in between the two 6-week treatment periods of patients with various ataxias. The primary outcome was absolute change in SARA.

Hundred and eight patients were randomized across Germany and Austria between January 2016 and February 2017, with a mean age of 54.8 years (SD 14.4), moderate disease (mean SARA 13.33 (SD 5.57)), and duration (median 10-year duration, *n* = 16, 14.8% had disease $\ge$ 20 years). 50.9% were female. The full analysis cohort included 80 patients with hereditary and 25 with non-hereditary or unknown cerebellar ataxia (total of 105) and was assessed in an intention-to-treat capacity. A mixed model for repeated measures was employed with fixed effects defined: treatment, visit, and treatment period.

There was no statistically significant difference in SARA scores between acetyl-dl-leucine and placebo (0.23, 95% CI: − 0.40, 0.85). Within each treatment period there appeared to be some evidence for a time effect (*p* = 0.04), with period estimate effect − 0.25 points (95% CI: − 0.50, 0.01; *p* = 0.06). However, the authors dismissed this as clinically insignificant in reference to the predetermined threshold of 1.5 points and further analysis was insignificant. Analysis of secondary outcome measures (including quality of life) also demonstrated no differences between the treatment group and placebo.

There were 246 AE reports with at least 86 patients having a single AE, which were similar between groups. The majority were mild (77.6%) with 3.3% considered serious. Only 4.9% of all AEs (acetyl-dl-leucine: *n* = 6, placebo: *n* = 2) were considered related to treatment.

### Comment

ALCAT failed to demonstrate effectiveness of acetyl-DL-leucine in a range of ataxias. This trial raises several issues: pre-specified subgroup analyses were acknowledged to be under-powered, and the range of ataxias may have masked any effect. Physical and speech therapies continued but do not appear to have been included as relevant factors.

JAMA Netw Open. 2021 Dec 1;4(12):e2135841*.*

## Motor and cognitive outcomes of cerebello-spinal stimulation in neurodegenerative ataxia

Previous work has shown cerebellar transcranial direct current stimulation (tDCS) to improve motor symptoms in neurodegenerative cerebellar ataxias possibly via improving neuroplasticity. This trial sought to investigate if repeated stimulation improved motor and cognitive outcomes in ataxia.

61 patients were recruited into a randomized, double-blind, sham-controlled trial, followed by an open-label phase in which all patients received tDCS. A variety of conditions were included the commonest being a genetic subgroup (39%); several aetiologies were excluded, e.g. trauma, stroke, or neoplastic; and various medications (sedatives, sodium-, and calcium-channel blockers) were stopped. The trial had two phases: sham-controlled and open-label. Stimulation consisted of five days a week for two-week periods (sham or real tDCS) from baseline and 12 weeks, with a subsequent 38-week period of regular follow-up with detailed assessments (some of which were via telemedicine due to the Covid-19 pandemic, 3.9%).

There was no association between stimulation type and patient perception (Cohen’s $\kappa$ = 0.03, *p* = 0.839) suggesting the sham stimulation was valid. Significant Time × Treatment interactions were relatively common across targets. That said, for motor outcomes in the placebo-controlled phase, comparing SARA in sham to tDCS a marginal mean difference of + 4.1 (95% CI: + 3.5, + 4.7) *p* < 0.001 was observed. The benefits were still seen in the open-label phase. For cognitive outcomes the Cerebellar Cognitive Affective Syndrome Scale (CCASS) had improvements (main mean scores − 7.0 (95% CI: − 10.4, − 3.5), *p* < 0.001) as well as reported quality of life (short-form health form 36, main mean scores: − 103.1 (95% CI: − 133.7, − 72.4), *p* < 0.001) with tDCS relative to sham, and baseline. There also appears to be an add-on effect, i.e. greater effect with two courses of tDCS than sham and tDCS. The data also revealed a negative correlation between improvement in SARA and baseline (${r}_{s}$ = − 0.64, *p* < 0.001) potentially favoring earlier intervention.

### Comment

Despite a variety of ataxias there was an improvement relative to sham-control and baseline. In addition, an add-on effect implies multiple courses of tDCS may be of benefit to patients with ataxia. Further work on ataxic subtypes will be required.

Brain. 2021 Sep 4;144(8):2310-2321

## Conclusion

This month’s journal club explored three recent trials in cerebellar ataxia with two negative and one positive outcome. These papers reveal lessons for future trials in cerebellar ataxia including population (single ataxia vs diverse range) and establishing baseline features such as physical and speech therapy intensity and quality as relevant factors, whilst pursuing potential DMTs in cerebellar ataxia.

